# Nanoparticle-directed and ionically forced polyphosphate coacervation: a versatile and reversible core–shell system for drug delivery

**DOI:** 10.1038/s41598-020-73100-5

**Published:** 2020-10-13

**Authors:** Werner E. G. Müller, Emad Tolba, Shunfeng Wang, Meik Neufurth, Ingo Lieberwirth, Maximilian Ackermann, Heinz C. Schröder, Xiaohong Wang

**Affiliations:** 1 ERC Advanced Investigator Grant Research Group at the Institute for Physiological Chemistry, University Medical Center of the Johannes Gutenberg University, Duesbergweg 6, 55128 Mainz, Germany; 2 Max Planck Institute for Polymer Research, Ackermannweg 10, 55128 Mainz, Germany; 3 Institute of Functional and Clinical Anatomy, University Medical Center of the Johannes Gutenberg University, Johann Joachim Becher Weg 13, 55099 Mainz, Germany

**Keywords:** Biochemistry, Biotechnology, Drug discovery, Diseases, Medical research

## Abstract

A drug encapsulation/delivery system using a novel principle is described that is based on an intra-particle migration of calcium ions between a central Ca^2+^-enriched nanoparticle core and the surrounding shell compartment. The supply of Ca^2+^ is needed for the formation of a coacervate shell around the nanoparticles, acting as the core of drug-loadable core–shell particles, using the physiological inorganic polymer polyphosphate (polyP). This polyanion has the unique property to form, at an alkaline pH and in the presence of a stoichiometric surplus of calcium ions, water-insoluble and stabile amorphous nanoparticles. At neutral pH a coacervate, the biologically active form of the polymer, is obtained that is composed of polyP and Ca^2+^. The drug-loaded core–shell particles, built from the Ca–polyP core and the surrounding Ca–polyP shell, were fabricated in two successive steps. First, the formation of the nanoparticle core at pH 10 and a superstoichiometric 2:1 molar ratio between CaCl_2_ and Na–polyP into which dexamethasone, as a phosphate derivative, was incorporated. Second, the preparation of the coacervate shell, loaded with ascorbic acid, by exposure of the Ca–polyP core to soluble Na–polyP and L-ascorbate (calcium salt). EDX analysis revealed that during this step the Ca^2+^ ions required for coacervate formation migrate from the Ca–polyP core (with a high Ca:P ratio) to the shell. Electron microscopy of the particles show an electron-dense 150–200 nm sized core surrounded by a less sharply delimited electron-sparse shell. The core–shell particles exhibited strong osteogenic activity in vitro, based on the combined action of polyP and of dexamethasone and ascorbic acid, which reversibly bind to the anionic polyP via ionic Ca^2+^ bonds. Drug release from the particles occurs after contact with a peptide/protein-containing serum, a process which is almost complete after 10 days and accompanied by the conversion of the nanoparticles into a coacervate. Human osteosarcoma SaOS-2 cells cultivated onto or within an alginate hydrogel matrix showed increased growth/viability and mineralization when the hybrid particles containing dexamethasone and ascorbic acid were embedded in the matrix. The polyP-based core–shell particles have the potential to become a suitable, pH-responsive drug encapsulation/release system, especially for bone, cartilage and wound healing.

## Introduction

Both biochemical/hormonal factors, like the bone morphogenetic proteins or growth hormones, and vitamins, like vitamin D^1^, as well as physical factors, like mechanical cues^2^, and the pH environment^3^ play pivotal functions during tissue regeneration around the healing bone. Focusing on the pH effect it has been established that an acidic pH environment induces autophagy in osteoblasts and bone resorption^4^. Based on available experimental finding it has been concluded that an elevated pH is beneficial for bone regeneration^3^. A slightly alkaline environment provides a “massive reserve of base”^4^ due to the high content of the alkaline salts of calcium, like calcium hydroxyapatite (HA) or calcium carbonate^5^. The alkaline reserve buffers metabolic H^+^ and by this inhibits osteoclast activity and bone resorption^6^. Conversely, at more alkaline pH conditions osteoblast formation is enhanced^3^. Under those conditions expression of the activating transcription factor^4^, a terminal regulator for osteoblasts^7^, as well as the dentin matrix acidic phosphoprotein^1^, a molecule involved in the progression of the osteoblasts into the osteocytes^8^, is upregulated. Finally, the alkaline phosphatase (ALP) activity requires slightly alkaline conditions (pH 7.4), while this enzyme is already inhibited at a pH of 6.9 ^4^. Therefore, implant(s) with functionally active molecules should be selected that respond adaptively to changes in pH. One of those molecules is the physiological inorganic polymer polyphosphate (polyP).

polyP is a polyanion that comprises a series of biochemically prominent properties. In animals/mammals this polyanion is synthesized as a polymer with a chain length of ~ 60 to 100 phosphate (P_i_) units that are linked together with energy-rich acid anhydride linkages^9^. Basically every mammalian cell synthesizes polyP, with the megakaryocyte/platelets as the dominant ones^10^. The major physiological features of polyP are, firstly, to provide metabolic energy after enzymatic cleavage by ALP^11–13^. Secondly, to act as a morphogenetically active polymer, especially for extracellular biochemical processes, like bone formation^14^, cartilage repair or teeth desensitization, as well as in wound healing^12^. Even more, polyP functions as a bioink for three-dimensional (3D) bioprinting of cell units^15^. Recently polyP came into focus when it could be elucidated that polyP, if exposed to divalent cations at near neutral pH conditions, condenses to a coacervate, a highly viscous gel-like material^16^. Coacervation results from interactions of oppositely charged polyions/ions in aqueous medium. These condensates are characterized by a low surface energy, high polymer concentration, highly viscous and dense but nonetheless fluidic properties^17^, as well as a by a distinguished bioavailability^16^. Recently it could be established that coacervates can also be applied in form of microcapsules for drug-delivery^18^.

The unique property of the polyP-controlled coacervation is its peculiarity to be guided by Ca^2+^ ions which are accumulated in the polyP-based amorphous Ca–polyP nanoparticles (Ca–polyP-NP), as determined by semi-quantitative EDX^19^, with a Ca to P atomic ratio of 0.84 or of 0.78, respectively, instead of a ratio of 0.5:1, assuming that the ionic reaction proceeds to completion. For the preparation of amorphous Ca–polyP-NP both a stoichiometric surplus of Ca^2+^ to P_i_ (in the polyP polymer) of ~ 2:1 and a pH milieu of 10 are required^20^. In contrast, for the formation of the polyP coacervate, which is the physiologically active form of polyP^16^, a pH of 7 has to be adjusted^12^. The nanoparticles are stable in distilled water in the absence of peptides or proteins. Only after exposure to peptides/proteins a transition of Ca–polyP-NP to a coacervate phase is seen due to a reduction of the zeta potential of the particle surface to near zero^16^. Additionally it has been demonstrated that cells embedded into the coacervate show a distinct migration and proliferation activity. These considerations indicate that polyP at higher pH (pH ~ 10) forms nanoparticles, while at nearly neutral conditions (pH ~ 7.4) a transition to the physiologically active polymer occurs^16^.

In the present study the property of Ca^2+^-polyP to shift from the amorphous nanoparticle state to the coacervate phase was utilized to fabricate trifunctional core–shell particles that exhibit strong osteogenic activity in vitro. The particles were formed from an amorphous Ca–polyP core into which dexamethasone (DEX) was embedded and a Ca–polyP coacervate shell with integrated ascorbic acid. These particles comprise a triple combination of bone anabolic components, (1) of polyP as a morphogenetically active polymer (see above), a property that is now increasingly highlighted by other groups as well [like: ref.^21^], (2) DEX, a synthetic glucocorticoid inducing osteogenic differentiation^22^, and (3) ascorbic acid, a natural water-soluble vitamin that promotes and facilitates osteogenic differentiation by increasing collagen type 1 secretion^23^. The two components, DEX and ascorbic acid, were immobilized via ionic, Ca^2+^-mediated electrostatic bonds between the polyanionic polyP and the (derivatized) DEX and ascorbic acid molecules. It is interesting to note that during formation of the Ca–polyP-coacervate shell which is fabricated around the Ca–polyP-NP core, the excess of Ca^2+^ trapped in the Ca–polyP core is extracted by the added soluble Na–polyP to form the stable Ca–polyP coacervate.

The described encapsulation technique opens a new avenue for a platform technology in which core nanoparticles are used that consist of a polyP-based scaffold to which bioactive molecules, e.g., anti-cancer drugs like daunorubicin, can be linked via ionic or electrostatic interaction^24^, and which are surrounded by a polyP-based coacervate that can be supplemented with an array of other anti-cancer compounds, even water-insoluble compounds like bortezomib^25^. The involvement of polyP in the nanoparticle fabrication also implicates the favorable function of polyP to activate the mechanistic target of rapamycin complex, which is modulated by the intracellular ATP level^26^. ATP is needed for the energy-consuming process of HA formation. Cellular ATP, metabolically synthesized from glucose, is required for the transition/development of osteoblasts from pre-osteoblasts^27^, while extracellular ATP, extracellular fuel, is generated enzymatically from polyP^11,13^. Besides of ATP, bone mineralization requires a slightly alkaline pH (7.4–7.6) at the osteon matrix^28^. During this process collagen fibrils are arranged in a quasi-hexagonal model assembly into which HA crystals are embedded^29^. The majority of the bone HA is deposited in the extracellular space, also driven by enzymes^30^, a process which requires metabolic energy as well, at least for the control of the transformation of amorphous calcium phosphate to HA^31^. The bulk of the extracellular ATP fuel most likely originates from polyP^13^.

The newly formed trifunctional core–shell particles were embedded into an alginate matrix and tested in vitro using SaOS-2 osteosarcoma cells^32^. In this system the polyP-based core–shell particles showed a marked induction of the mineralization process, qualifying them as a novel intelligent material which is adaptable to environmental changes for further clinic-oriented development. This material is expected to be applied after mandibular/jaw augmentation operations.

## Materials and methods

### Materials

Na–polyphosphate (Na–polyP) with an average chain length of 40 phosphate units was purchased from Chemische Fabrik Budenheim (Budenheim; Germany).

### Preparation of the dexamethasone-free Ca–polyP nanoparticles

As initially introduced^20^ Ca–polyP nanoparticles (Ca–polyP-NP) were prepared from a 2:1 molar ratio between CaCl_2_ and Na–polyP (based on phosphate). In brief, 2.8 g of CaCl_2_·2H_2_O (#223506, Sigma-Aldrich, Taufkirchen; Germany) were dissolved in 25 mL of distilled water and added dropwise to a Na–polyP solution (1 g in 25 mL of water) at room temperature and a pH of 10. After stirring for 3 h the particles were collected by filtration, washed twice with water and freeze-dried at − 80 °C. The spherical nanoparticles, termed “Ca–polyP-NP”, had a size of between 50 and 80 nm in diameter.

### Preparation of the dexamethasone loaded Ca–polyP nanoparticles

DEX was loaded into the Ca–polyP-NP at different mass concentrations, at 5 and 10 wt% with respect to Na–polyP. Solid 0.05 g [or 0.1 g] of dexamethasone 21-phosphate disodium salt (DEX-P; #D1159 Sigma) was mixed with 1 g of Na–polyP and dissolved in 50 mL of distilled water at a pH of 10 (adjusted with 0.5 M NaOH). Then, 2.8 g of CaCl_2_·2H_2_O, dissolved in 50 mL of water (pH 10), were dropwise added over a 30 min period at room temperature and then allowed to stand for 3 h. Subsequently, the particles were collected, washed 3-times with water and freeze-dried. The nanoparticles were termed “Ca–polyP/D-NP” (5 wt% DEX containing) and “Ca–polyP/D10-NP” (10 wt% DEX containing).

### Preparation of the core–shell particles

“Ca–polyP/D-NP” were coated with an ascorbic acid-polyP coacervate shell as follows. A solution of 10 mL of Tris hydrochloride (1 M; #10812846001, Sigma; pH adjusted to 7.4) was prepared which contained 0.1 g of “Ca–polyP/D-NP”. To this suspension a solution of 0.05 g of Na–polyP, dissolved in 10 mL of Tris–HCl (1 M; pH 7.4), was added (duration of 10 min). After centrifugation, the core–shell particles were suspended in 10 mL of a solution containing 0.17 g of calcium l-ascorbate dihydrate (#359645, Sigma). The suspension formed was gently stirred for 30 min at pH 7.4. Then the particles were collected, washed with water and freeze-dried. The core (DEX containing)-shell (ascorbate enriched [AA]) particles were termed “Ca–polyP/D-NP@polyP/AA-Coa”.

In a separate series the core–shell particles were prepared in the same way with “Ca–polyP-NP” core nanoparticles which were covered with polyP coacervate supplemented with calcium l-ascorbate dihydrate; “Ca–polyP-NP@polyP/AA-Coa”.

### X-ray diffraction

The samples were analyzed by X-ray powder diffraction (XRD) with a D8 Advance A25 diffractometer (Bruker, Billerica; MA) connected with a monochromatic Cu-Kα radiation. Dried powder samples were used.

### EDX analysis

The EDX (energy dispersive X-ray spectroscopy) experiments were run with an EDAX Genesis EDX detector, connected with a scanning electron microscope (Nova 600 Nanolab, FEI, Eindhoven; The Netherlands). For the analyses a collection time of 30–45 s at 10 kV was applied. Section of 5 to 10 μm^2^ were chose for the determination. In the semi-quantitative approximation, the signals, corresponding to the selected elements, were quantitated^33^. The results showed an error of ~ 10%.

### Microscopic analysis

A Zeiss Gemini 1530 (Zeiss Oberkochem; Germany) was applied for capturing high-resolution SEM (scanning electron microscope) images. For the lower magnifications, an ESEM (environmental scanning electron microscope) with an ESEM XL-30 microscope (Philips, Eindhoven; Netherlands) was used. Samples for electron microscopic SEM/ESEM analyses were fixed in 2% [v/v] aqueous glutaraldehyde fixative and finally treated with osmium oxide. Then, the specimens were processed through acetone dehydration steps and subjected to critical point drying at 43 °C. The TEM (transmission electron microscope) analyses were performed with a TemCam-F416 (4 K × 4 K) CCD camera (TVIPS, Gauting; Germany) hooked to a Tecnai 12 transmission electron microscope (FEI, Eindhoven; The Netherlands), by using an accelerating voltage of 120 kV.

A digital light microscope VHX-600 (Keyence, Neu-Isenburg; Germany) with a VH-Z25 zoom lens was applied for light microscopic inspections.

### Cells and incubation conditions

SaOS-2 cells (human osteogenic sarcoma cells)^34^ were purchased (#89050205; Sigma) and cultured in McCoy’s medium (containing CaCl_2_; 1 × 10^−3^ M; #M4892, Sigma) with 5% heat-inactivated fetal calf serum (FCS; #A3840001, Thermo Fisher Scientific; Waltham, MA), l-glutamine (2 × 10^−3^ M) and gentamicin (50 μg mL^−1^) and incubated in 25 cm^2^ flasks or 24-well cell culture plates (Thermo Fisher Scientific). Routinely, the cells were seeded at a density of 2 × 10^4^ cells per 3 mL well and cultivated for 3–5 days in medium/FCS.

### Growth of SaOS-2 cells onto the hydrogel

The hydrogel was prepared with Na-alginate (#W201502; Sigma-Aldrich) as the basis. A 3% (w/w) alginate solution in McCoy’s medium, containing gentamicin sulfate (15 µg mL^−1^; #G1914, Sigma-Aldrich), was composed^30^. After stirring to homogeneity and subsequent centrifugation (500 × *g*; 3 min) a 100 µL aliquot was layered into every well of a 24-well cell culture plate, followed by a short exposure to 100 µL of 2.5% (w/w) CaCl_2_·2H_2_O in physiological saline (#P3813, Sigma) for 5 min. After this step the supernatant fluid was pipetted off and substituted with 300 µL of McCoy’s medium/5% FCS. Subsequently, the medium/serum was removed and the gel was overlayed with 100 µL of SaOS-2 cells (5 × 10^5^ cells mL^−1^) in McCoy’s medium/5% FCS. Cultivation was routinely for 3 days followed by the MTT test. In one series, the matrix alginate was supplemented with 50 µg mL^−1^ of “Ca–polyP-NP”, and the growth of cells was determined time-dependently for 1 day, 2 days, 3 days, and 5 days. In parallel different concentrations of Ca–polyP-NP were added and growth was determined after 3 days.

Finally, and were shown, the alginate cultures were supplemented with 50 µg mL^−1^ (or 100 µg mL^−1^) of “Ca–polyP-NP”, “Ca–polyP/D-NP”, “Ca–polyP-NP@polyP/AA-Coa”, or “Ca–polyP/D-NP@polyP/AA-Coa” and the cell viability was determined.

### Cultivation of SaOS-2 cells within the hydrogel

In this experiment 100 µL of SaOS-2 cells (5 × 10^5^ cells mL^−1^) in McCoy’s medium/5% FCS were pipetted into each well (24-well plate). Then the cells were overlayed with 100 µL of 4% (w/w) alginate solution in McCoy’s/FCS and the assays were gently mixed. After incubation for 60 min the gel was covered with 100 µL of 2.5% (w/w) CaCl_2_·2H_2_O in physiological saline for 5 min. After this the saline was pipetted off from the alginate layer which was subsequently washed twice with McCoy’s medium/5% FCS and finally covered with 300 µL of McCoy’s/FCS. In the series for testing the extent of mineralization the alginate gel was enriched with 100 µg mL^−1^ (final concentration) of “Ca–polyP-NP”, “Ca–polyP/D-NP”, “Ca–polyP-NP@polyP/AA-Coa”, or “Ca–polyP/D-NP@polyP/AA-Coa”. The incubation was for 5 days.

### MTT viability assay

The growth rate of the cells based on the viability test the colorimetric MTT (thiazolyl blue tetrazolium bromide; # M2128, Sigma) assay. The gel with the cells was incubated first with MTT (1 µg mL^−1^; 2 h) and subsequently with 20% SDS in 50% dimethyl-formamide (#227056, Sigma) for 24 h. The formazan grains were dissolved and the optical density was measured at 595 nm.

### DEX and ascorbic acid encapsulation efficiency and in vitro release

Dexamethasone phosphate (DEX-P) release was measured via ultraviolet–visible spectroscopy (UV–Vis spectrometer; NanoDrop 2000c; Thermo Fisher Scientific). Firstly, a calibration curve was prepared with DEX-P by measuring the absorbance values at 242 nm of different concentrations (0–20 mg mL^−1^) to calculate the amount of DEX-P release^35^. For determination of the release kinetics 20 mg of the DEX-P were loaded into “Ca–polyP/D-NP” and then dissolved in 1 mL of HCl (0.5 M), followed by dilution with 4 mL of water while vigorously vortexing for 10 min and then centrifugation at 3000 rpm for 10 min. The absorbance of the clear solution obtained was measured in the UV–Vis spectrometer and the concentration was calculated based on the calibration curve. The drug, DEX-P, encapsulation efficiency (EE) from the “Ca–polyP/D-NP” was calculated as the percentage ratio between the entrapped amount of DEX-P in NPs, after dissolution of the “Ca–polyP/D-NP” and the initial amount of DEX-P added during the synthesis of the nanoparticles using the following equation: EE = [the encapsulated amount of DEX-P (mg)]: [the initial amount DEX-P] × 100. The release experiments were performed in fivefold parallel runs and the cumulative profiles are given.

The encapsulation efficiency as well as the in vitro release of ascorbic acid was likewise determined UV spectroscopically at 244 nm. The particles were again dissolved in HCl (0.1 M; pH 3). The samples were centrifuged at 3000 × *g* (10 min) and the ascorbic acid concentrations was determined in the supernatant applying the iodine titration/potentiometric titration technique; the cumulative profiles are given^36^. Even though ascorbic acid is considered to be most stabile at pH 3, a second method was applied for the determination of the ascorbic acid concentration which is based on the reduction of 2,6-dichloroindophenol sodium salt hydrate (# D1878, Sigma) by ascorbic acid^37^. The ascorbic acid solution was supplemented with 2.5% metaphosphoric acid and read at 520 nm spectrophotometrically. After construction of the calibration curve the concentration was obtained.

### Mineralization assay

SaOS-2 cells were incubated within the alginate gel under the conditions described above. After termination of the cultivation the medium/FCS was carefully removed and the cells were stained with 10% Alizarin Red S (#A5533, Sigma-Aldrich) as described^38,39^. In parallel, the assays were quantitatively assessed for the extent of ossification by applying the Alizarin Red S spectrophotometric assay^14^. The amount of bound Alizarin Red S is given in µmoles. Values were normalized to total DNA in the samples^40^.

### Statistical analysis

After verification that the respective values follow a standard normal Gaussian distribution and the variances of the respective groups are equal, the results were statistically assessed using the independent two-sample Student’s *t*-test^41^.

## Results

### Preparation of the core–shell nanoparticles/coacervate

**Figure 1:**
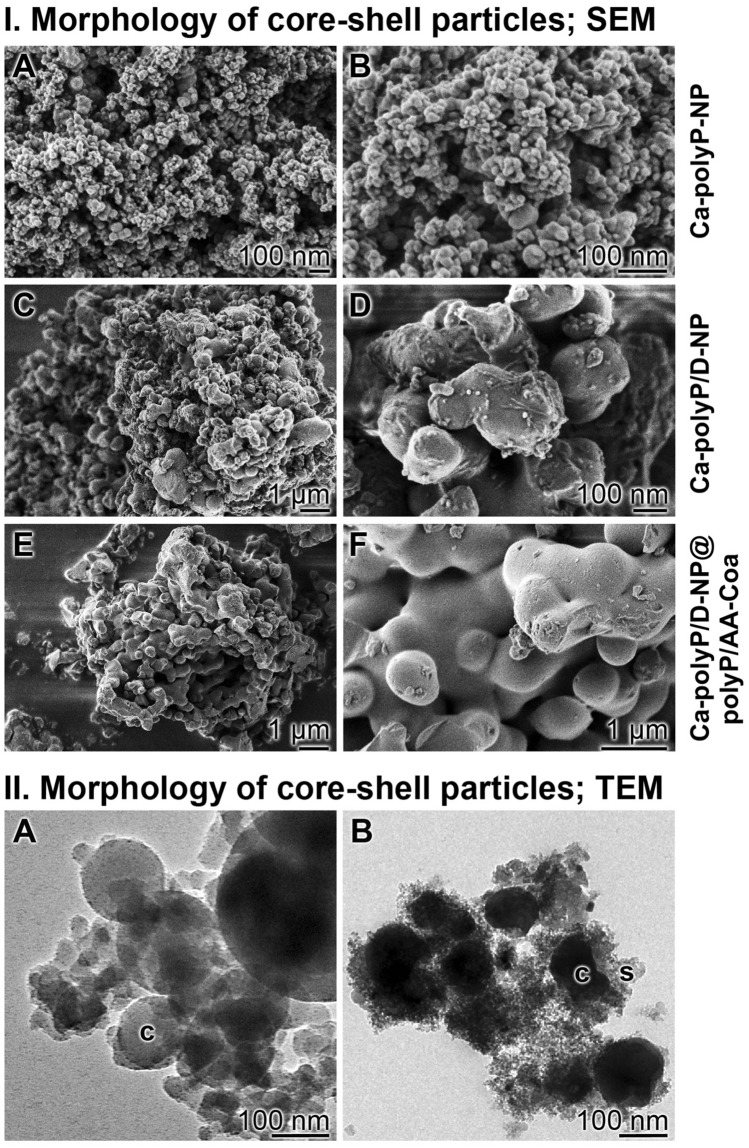
Analysis of the core–shell nanoparticles. (**I)** Morphology of the core–shell particles; SEM. Both the morphology of their nanoparticle core, (**I-A** and **I-B**) “Ca–polyP-NP”, without or (**I-C** and **I-D**) with integrated dexamethasone, “Ca–polyP/D-NP”, and (**I-E** and **I-F**) of the complete “Ca–polyP/D-NP@polyP/AA-Coa” particles, containing dexamethasone and ascorbic acid, are shown. (**II)** Electron microscope images of (**II-A**) the drug (dexamethasone) loaded core nanoparticles, “Ca–polyP/D-NP” and (**II-B**) the drug (dexamethasone and ascorbic acid) loaded core–shell nanoparticles, “Ca–polyP/D-NP@polyP/AA-Coa”; *c* core, *s* shell. High-resolution TEM.

The dexamethasone-free Ca–polyP nanoparticles, ‘‘Ca–polyP-NP’’, and the dexamethasone loaded Ca–polyP nanoparticles, “Ca–polyP/D-NP”, were prepared by using a 2:1 molar ratio between CaCl_2_ and Na–polyP (based on the monomer). The drug-free nanoparticles, “Ca–polyP-NP”, were loaded with DEX, the phosphate-derivative dexamethasone 21-phosphate (DEX-P) was used, with 5 or 10 wt%, respectively, in relation to Na–polyP added. The diameter of the spherical particles of “Ca–polyP-NP” measured between 50 and 80 nm (average of 83 ± 17 nm; 20 determinations); Fig. 1I-A and I-B. In contrast, the particles loaded with 5 wt% [10 wt%] DEX-P, “Ca–polyP/D-NP” [“Ca–polyP/D10-NP”], are considerably larger with an average of 296 ± 105 nm for both preparations; the “Ca–polyP/D-NP” are shown in Fig. 1I-C and I-D.

Both layers of the core–shell particles are formed of polyP which has been stabilized by Ca^2+^. While the core is prepared first at pH 10 resulting in the fabrication of nanoparticles from polyP and Ca^2+^ (“Ca–polyP-NP” or “Ca–polyP/D-NP”), the shell is secondarily synthesized from the starting polymer Na–polyP. Since the nanoparticles are formed at super-stoichiometric conditions between calcium and phosphate (see next paragraph) the shell is layered around them by a supply of Na–polyP only (without CaCl_2_). During the coacervate formation the required Ca^2+^ ions are translocated from the Ca–polyP-NP or Ca–polyP/D-NP core to the shell. The morphology of the “Ca–polyP/D-NP@polyP/AA-Coa” particles, consisting of a DEX containing core and an ascorbate [AA] enriched shell, was visualized by SEM (Fig. 1I-E, I-F) and TEM (Fig. 1IIB). In TEM, the core of the nanoparticles, “Ca–polyP/D-NP” (Fig. 1IIA), appears as electron-dense material of a size of 150–200 nm. The centers of the particles comprise both bright and darker delimited areas. The core–shell particles, “Ca–polyP/D-NP@polyP/AA-Coa”, also display the sharply defined electron-dense core around which a more fluffy and less clearly delimited electron-sparse layer has been formed (Fig. 1IIB).

Like in the initial study, reporting that the Ca–polyP nanoparticles are in an amorphous phase^20^, also the particles prepared in the present contribution have been subjected to XRD analysis. Likewise the “Ca–polyP-NP”, “Ca–polyP/D-NP” as well as the “Ca–polyP/D-NP@polyP/AA-Coa” samples showed an XRD pattern characteristic for an amorphous phase (data not shown).

### Element distribution within the particles

**Figure 2:**
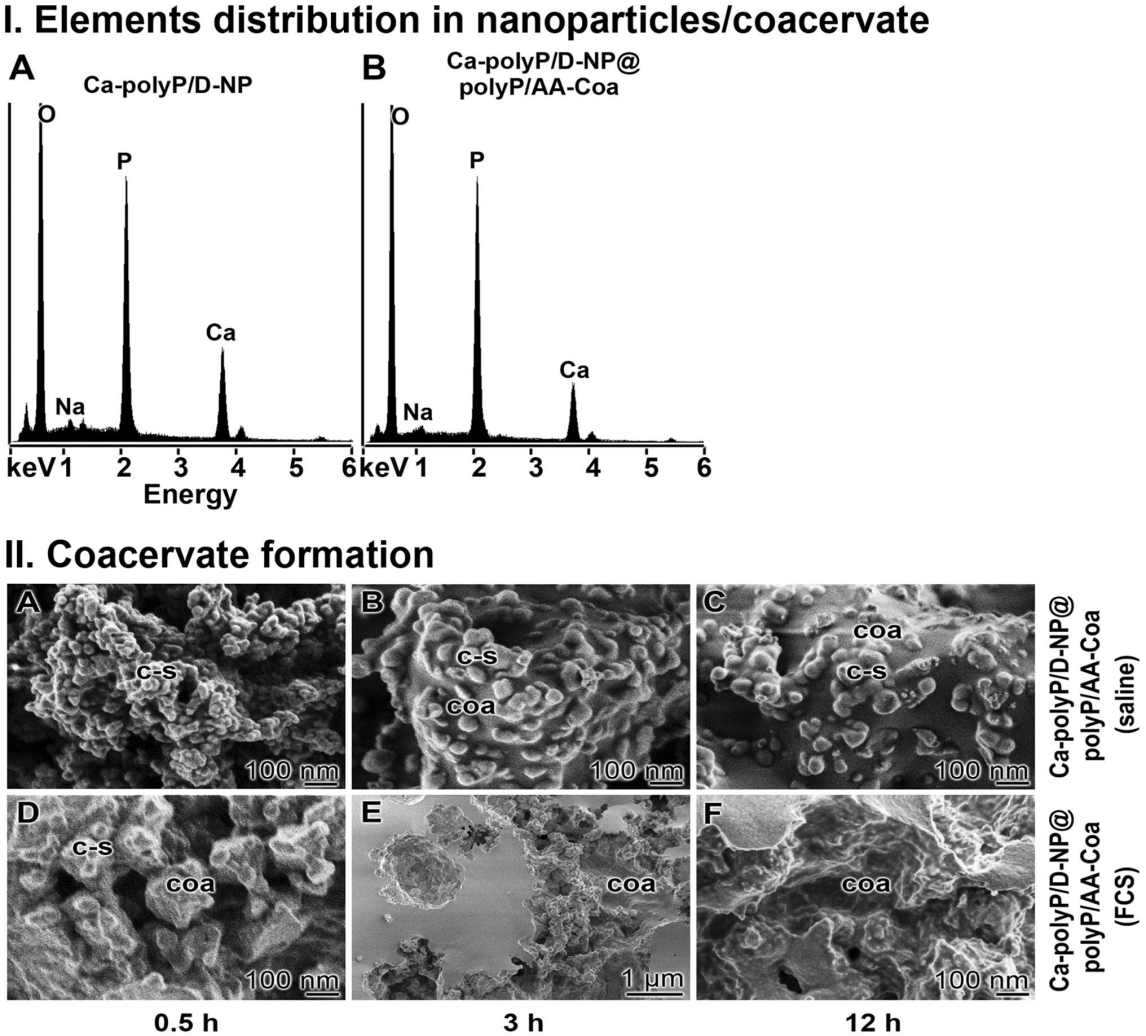
Coacervate formation. (**I**) Element re-distribution of calcium and phosphate during the coacervate shell formation around the Ca–polyP core, resulting in a stoichiometric Ca:P ratio of ≈0.5, as revealed by semi-quantitative EDX. (**I-A**) In the core of the “Ca–polyP/D-NP”, a Ca:P ratio of 0.83 is measured, while (**I-B**) the core–shell particles, “Ca–polyP/D-NP@polyP/AA-Coa”, show an overall ratio of 0.47. (**II**) Kinetics of transformation of the core–shell particles, “Ca–polyP/D-NP@polyP/AA-Coa”, including their nanoparticle core, to a coacervate in (**II-A** to **II-C**) 0.9% saline *versus* (**II-D** to **II-F**) 0.9% saline supplemented with 2% FCS; SEM. The study was performed at room temperature. After termination of the incubation period the samples were dried and inspected. It is seen that the core–shell particles only slowly undergo coacervation in 0.9% saline, in contrast to the particles suspended in 2% FCS. During the incubation period of 12 h the core–shell particles (c–s) transform almost totally to the coacervate (coa) if they are kept in 2% FCS.

Using the semi-quantitative EDX approach a shift of the distribution between calcium and phosphorous was determined between the nanoparticles and the nanoparticle/coacervate core–shell samples (Fig. 2I). The EDX spectral analysis of the “Ca–polyP/D-NP” sample revealed a Ca:P ratio of 0.83 (Fig. 2IA), while the spectroscopy of the “Ca–polyP/D-NP@polyP/AA-Coa” particles showed an element ratio of 0.47 (Fig. 2IB). This shift reflects a re-distribution of the elements from an over-stoichiometric ratio in the “Ca–polyP/D-NP” core to a close to stoichiometric ratio in the “Ca–polyP/D-NP@polyP/AA-Coa” preparation. This shift is taken as an indication that the Ca^2+^ becomes redistributed during the coacervate shell formation to an overall stoichiometric ratio of ≈ 0.5 between calcium and phosphate.

### Transformation of the core–shell particles to a coacervate

In a previous study we reported that polyP nanoparticles^16^ have on their surface a high zeta potential, preventing them to form aggregates. However, after an exposure to peptides/proteins the zeta potential drops, allowing a collision of the particles and coacervate formation. This process is also seen for the DEX/AA core–shell particles, the “Ca–polyP/D-NP@polyP/AA-Coa”.

Suspending the “Ca–polyP/D-NP@polyP/AA-Coa” core–shell particles into 0.9% saline (1 g of nanoparticles into 50 mL of saline) shows a very slow transformation of the particles into a coacervate (Fig. 2IIA–C). One half h after suspending the borders of the core–shell particles are still distinctly seen (Fig. 2IIA), while after a prolonged incubation period first patches of the rubber-like coacervate with a smooth surface are found that have been formed around the particles (Fig. 2IIB, C). However, if the core–shell particles are suspended 2% fetal calf serum (FCS) (Fig. 2IID–F) a rapid transformation of the particles into the coacervate occurs. The first clear signs of coacervation are seen already after 0.5 h (Fig. 2IID).

### Loading efficiency and release kinetics from the particles

The polyP core particles were loaded with DEX-P (5 or 10 wt%) by starting with Na–polyP and CaCl_2_·2H_2_O at pH 10. After termination of the reaction and washing the particles a content of 2.8 ± 0.4 wt% for the “Ca–polyP/D-NP” (5 wt% DEX containing) and of 3.5 ± 0.6 wt% for the “Ca–polyP/D10-NP” formulation (5 parallel experiments) were determined.

In parallel, the yield of ascorbic acid in the core–shell particles, “Ca–polyP/D-NP@polyP/AA-Coa”, was determined by applying the titration techniques as described under “Materials and methods”. The content of calcium l-ascorbate dihydrate in the particles was 12.4 ± 1.7 wt%.

**Figure 3:**
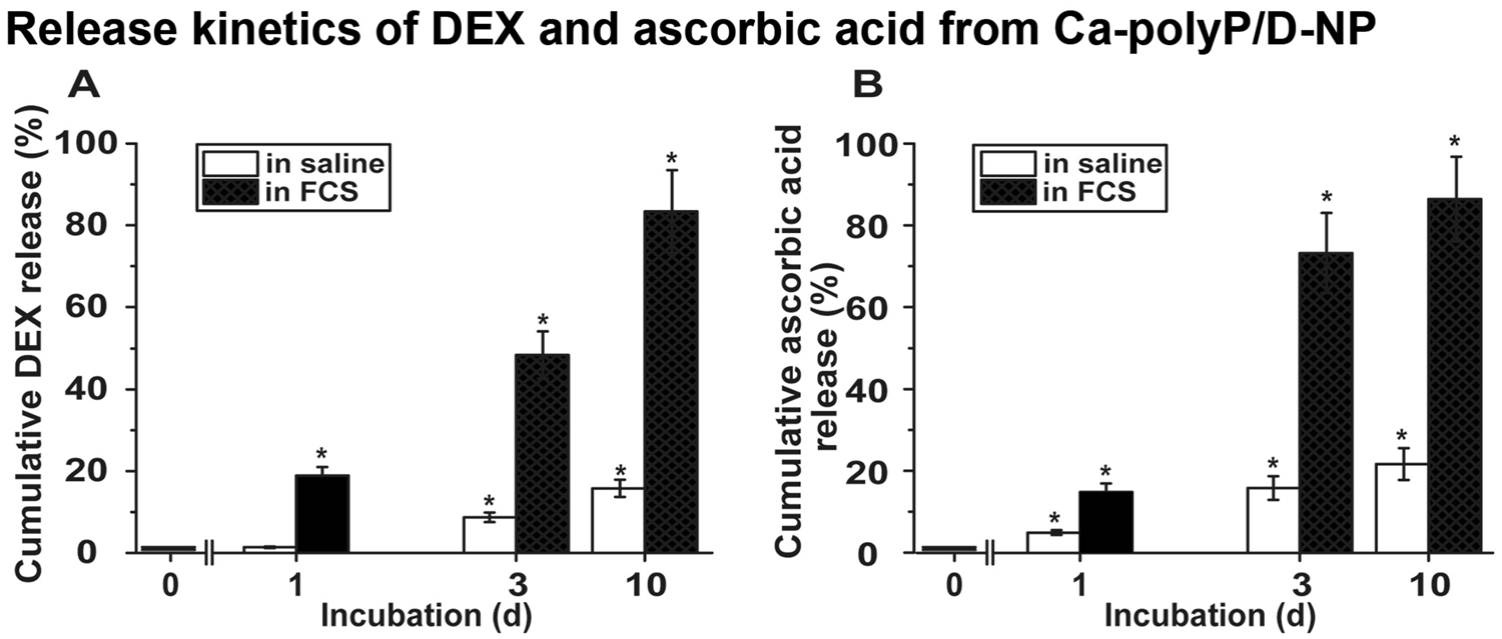
Release kinetics of DEX from “Ca–polyP/D-NP” particles and ascorbic acid from “Ca–polyP/D-NP@polyP/AA-Coa” core–shell particles in saline or in saline supplemented with 2% FCS. (**A**) The concentration of DEX was determined spectrophotometrically, while (**B**) the liberated ascorbic acid was quantified by titration. The means ± SD of five independent experiments are given; (*, *p* < 0.005).

The drug (DEX and ascorbic acid) release from the particles was determined by suspending the particles, “Ca–polyP/D-NP”, or the core–shell nanoparticles/coacervate, “Ca–polyP/D-NP@polyP/AA-Coa”, either in saline or in 2% FCS (in saline) for up to 10 days (Fig. 3). The cumulating release data show that initially the release of DEX (Fig. 3A) and ascorbic acid (Fig. 3B), after 1 day, is almost the same for both drugs. However, after an incubation for 3 days the release of ascorbic acid in 2% FCS is significantly higher, with 73.2 ± 9.8%, compared to the release of DEX (48.4 ± 5.8%) during the same period. In this series the determination of ascorbic acid was performed after dissolution of the particles in HCl (pH 3). To rule out any potential variance due to decomposition, a determination with 2,6-dichloroindophenol was applied. With this method values have been measured that are not significantly different from the results obtained with the first spectrophotometric assay. After a prolonged incubation for a 10 days period the DEX release from “Ca–polyP/D-NP” is 83.4 ± 10.1% and similarly high as for ascorbic acid (86.4 ± 10.4%) from the “Ca–polyP/D-NP@polyP/AA-Coa” particles.

During the incubation, especially if added to 2% FCS, the particles lose their individual roundish morphology and after 2–3 days they show distinct signs of coacervation. The NP fuse and have an appearance as those shown in Fig. 2IID–F.

### Growth of SaOS-2 cells onto the hydrogel

SaOS-2 cells were seeded either onto the alginate hydrogel or grown within the hydrogel (see below), in the absence of polyP or together with the different polyP formulations.

**Figure 4:**
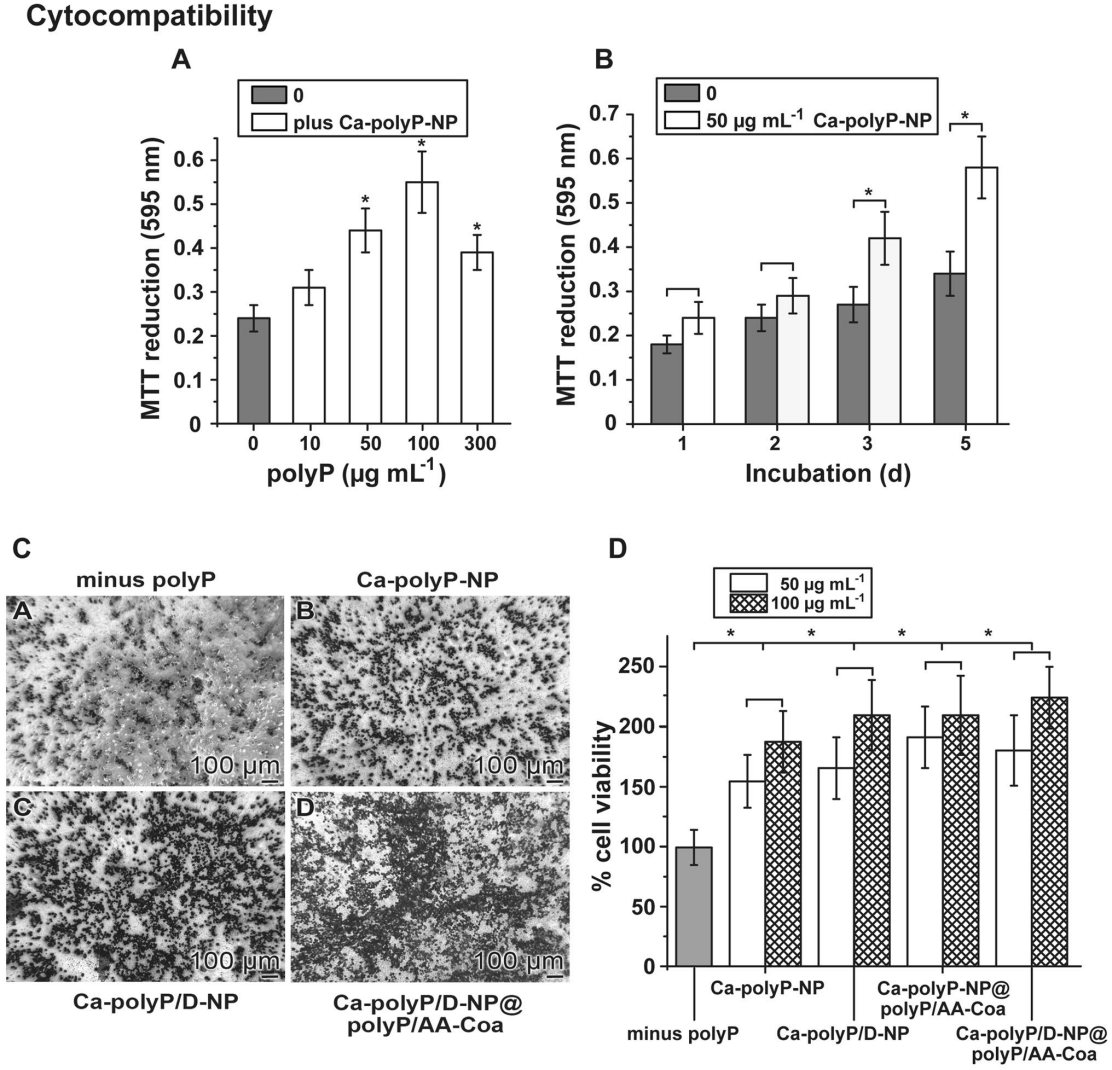
Cytocompatibility of the matrices. Effect of the polyP formulations on the growth of SaOS-2 cells onto polyP-free or polyP-supplemented alginate hydrogels. The Na-alginate hydrogels were hardened with CaCl_2_. (**A**) Growth of the cells onto the hydrogel without (0 µg mL^−1^ polyP) or with increasing concentrations of “Ca–polyP-NP”. The metabolic activity is measured with the MTT assay and the absorbance is given as A_595nm_ units. (**B**) Time-dependent effect of 50 µg mL^−1^ of “Ca–polyP-NP” on the activity of cells, in comparison to the one after exposure in the absence of polyP. (**C**) Effect of polyP on the density of cells gown onto different matrices. The cells were seeded onto the alginate gel in the absence of polyP (C[A]) or onto alginate supplemented with 100 µg mL^−1^ of (C[B[) “Ca–polyP-NP”, of (C[C]) “Ca–polyP/D-NP” or of (C[D]) “Ca–polyP/D-NP@polyP/AA-Coa”. The microscopic image were taken after an incubation period of 3 days. (**D**) Growth/viability of the cells (given as % of the control [minus polyP]) after an incubation of 3 d, as determined by the MTT assay. The cells were layered on the hardened alginate matrices and incubated for 3 days in the well plates. Alginate remained either polyP-free, or was supplemented with polyP as indicated, selecting 50 µg mL^−1^ or 100 µg mL^−1^ of “Ca–polyP-NP”, of “Ca–polyP/D-NP”, of “Ca–polyP-NP@polyP/AA-Coa”, or of “Ca–polyP/D-NP@polyP/AA-Coa”. Data represent means ± SD of ten independent experiments; the significances between the control (without polyP) and the respective polyP formulations were calculated (*, *p* < 0.005).

In the experiment shown in Fig. 4-A, the cells were grown onto the alginate hydrogel, supplemented with increasing concentrations of “Ca–polyP-NP”. The hydrogel from Na-alginate was hardened to a gel-like matrix with CaCl_2_ as described under “Materials and methods”. Then, cells were overlayed onto the gel and incubated for 3 days. The cell viability was determined with the MTT assay by which the cellular metabolic activity as an indicator of cell viability is assessed. Addition of 10 µg mL^−1^ of “Ca–polyP-NP” to the hydrogel results already in a slight increase in metabolic viability with an absorbance value (at 595 nm) of 0.31 ± 0.04, in comparison to the gel lacking polyP (A_595nm_ 0.24 ± 0.03). Increasing the polyP concentration to 50 µg mL^−1^ results in a significant increase of the absorbance value to 0.44 ± 0.05 and at 100 µg mL^−1^ even to 0.55 ± 0.07. Increasing further the concentration to 100 µg mL^−1^ reduces the activity to A_595nm_ 0.39 ± 0.04. In a time-dependent analysis, selecting 50 µg mL^−1^ of “Ca–polyP-NP”, a significant difference in the absorbance value between the polyP-free assay and the polymer-containing assay becomes significant after 3 days (Fig. 4B).

For the comparative testing of the different hydrogels SaOS-2 cells were grown onto those matrices for 3 days and then inspected by light microscopy (Fig. 4C). In the absence of polyP the cells were loosely scattered on the gel (Fig. 4C[A]). If polyP in form of “Ca–polyP-NP” was added onto the alginate a significant higher density of cells was recorded (Fig. 4C[B]) which even increased when the cultures were grown onto “Ca–polyP/D-NP” (Fig. 4C[C]) or onto “Ca–polyP/D-NP@polyP/AA-Coa” (Fig. 4C[D]).

The quantitative, comparative assessment of the viability/growth of the SaOS-2 cells on the different matrices was performed with the MTT assay (Fig. 4D). In the absence of polyP the cells gave an OD of the reduced insoluble purple formazan of 0.27 ± 0.04 OD units, this value was set to 100%. The percent cell viability in the assay with “Ca–polyP-NP” at concentrations of 50 µg mL^−1^ (or of 100 µg mL^−1^) increased, after an incubation period of 3 days, significantly to 156 ± 22% (188 ± 26%). Supplementation of the particles with polyP, DEX or with ascorbic acid only slightly, but not significantly increased the growth of the cells to like to 192 ± 26% (211 ± 33%) for the samples with “Ca–polyP-NP@polyP/AA-Coa”.

### Cultivation of SaOS-2 cells within the hydrogel: mineralization

**Figure 5:**
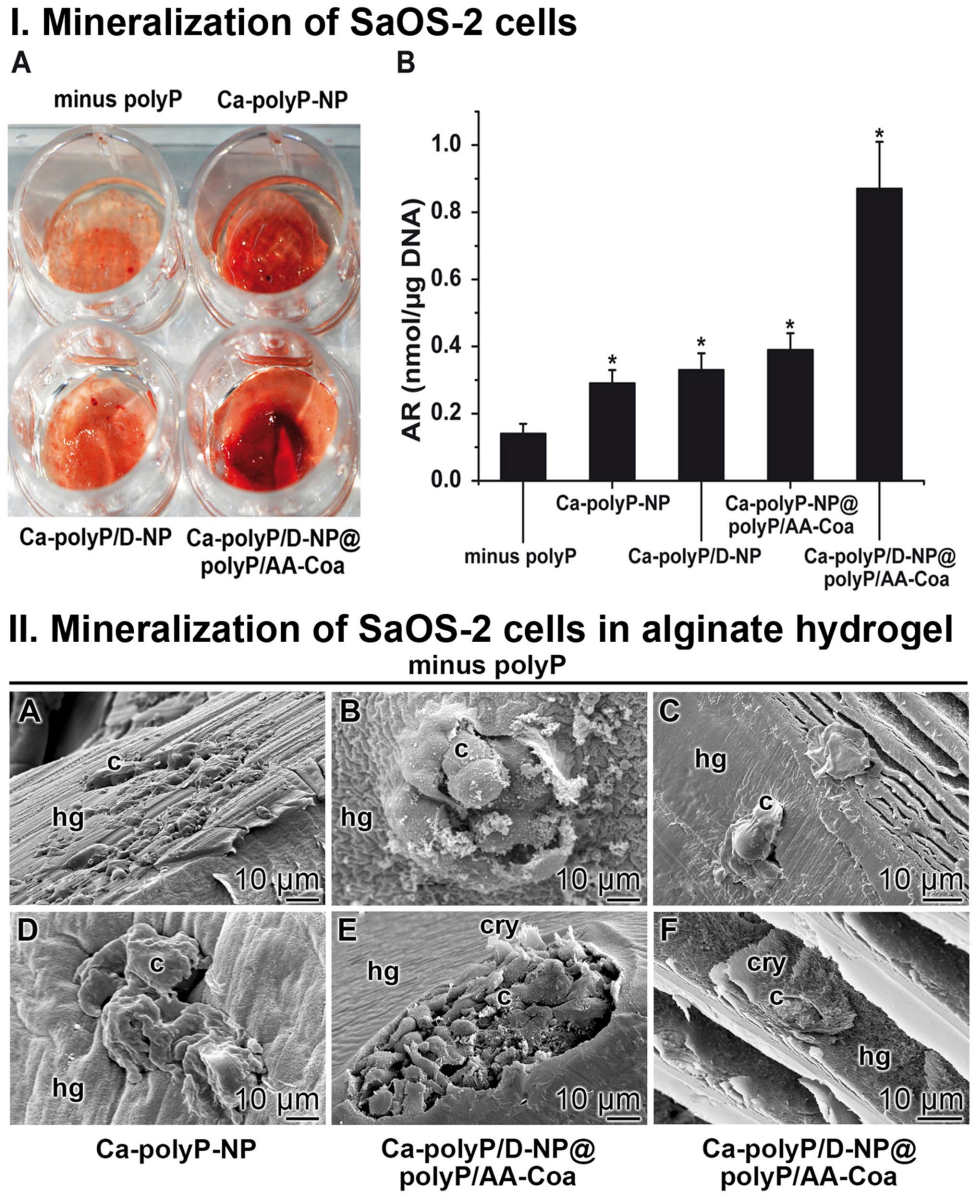
Effect on cell surface mineralization. **I.** Mineralization of SaOS-2 cells within an alginate hydrogel supplemented with different formulations of polyP. (**I-A**) The cells were embedded into the alginate either in the absence of polyP (minus polyP) or into a matrix containing either “Ca–polyP-NP” or the DEX-enriched particles, “Ca–polyP/D-NP”, as well as “Ca–polyP/D-NP@polyP/AA-Coa”. At the end of incubation (5 d) the gel was colored with Alizarin Red S. (**I-B**) Quantitative assessment of mineralization onto SaOS-2 cells, embedded into the hydrogel, either in the absence of the polymer (minus polyP), or in the presence of the polymer in the form of “Ca–polyP-NP”, “Ca–polyP/D-NP”, “Ca–polyP-NP@polyP/AA-Coa” or “Ca–polyP/D-NP@polyP/AA-Coa”, as described under “Materials and methods”. The determinations were performed after 5 days with Alizarin Red S. The signals determined were normalized to allow correlation with the cell numbers. Means ± SD; *n* = 10_; *,_ *p* < 0.005). **II.** Visualization of SaOS-2 cells within the alginate-based hydrogel. The cells were cultivated within the hydrogel (**A** to **C**) in the absence of polyP, (**D**) in “Ca–polyP-NP”-enriched gel, or (**E** and **F**) in “Ca–polyP/D-NP@polyP/AA-Coa”-enriched gel; ESEM. The cells were seeded into the respective hydrogel (hg) and incubated for 5 days. The cells (c) and crystallites (cry) on their surfaces are marked.

In a second series of experiments the SaOS-2 cells were embedded into an alginate hydrogel which was hardened by a subsequent hardening step using CaCl_2_. After an incubation period for 5 days followed by a staining process with the mineralization indicator Alizarin Red S only a slight red coloring of the hydrogel becomes visible (Fig. 5IA). In contrast, if the hydrogel is substituted with 100 µg mL^−1^ of polyP, administered as “Ca–polyP-NP”, an already more intense red staining becomes obvious. After including DEX into the particle formulation (“Ca–polyP/D-NP”) no significant change becomes overt. However, if ascorbic acid is additionally included into the particles, more specific into the coacervate layer that is covering the DEX-containing particles, the “Ca–polyP/D-NP@polyP/AA-Coa” hybrid formulation elicits an intensive red staining reflecting a full scale mineralization process.

A quantitative assessment of the increasing mineralization was accomplished by application of the Alizarin Red S spectrophotometric detection system (Fig. 5IB). The results unambiguously show that in the absence of polyP only a low absorbance developed by the cells in the hydrogel (0.14 ± 0.03 nmoles of dye incorporated into cellular DNA normalized to 1 µg) which reflects only a marginal mineralization scale. However, addition of 100 µg mL^−1^ of “Ca–polyP-NP” into the gel increased significantly the extent of Alizarin Red detectable mineralization to a value of 0.29 ± 0.04 nmoles µg^−1^. While a further enrichment of the particles with DEX only marginally increased the mineralization (0.33 ± 0.05 nmoles µg^−1^) the addition of ascorbic acid, deposited as coacervate around the DEX-free polyP-based nanoparticles (“Ca–polyP-NP@polyP/AA-Coa”), had a significantly stronger enhancing effect to 0.39 ± 0.05. However, if all three components present in an osteogenic differentiation cocktail, phosphate (as polyP), DEX and ascorbic acid, were included in the particles, like in “Ca–polyP/D-NP@polyP/AA-Coa”, a very intense SaOS-2-caused mineralization developed (0.87 ± 0.12 nmoles µg^−1^). Based on this finding we conclude that the hybrid particles “Ca–polyP/D-NP@polyP/AA-Coa” elicit in a condensed application form a drug (DEX—ascorbic acid) delivery system, suitable for the induction of a localized mineralization process.

### In situ mineralization of Saos-2 cells within the hydrogel

A direct visualization of the mineral deposits onto the SaOS-2 cells within the alginate hydrogel could be performed by ESEM. The cells were embedded within the hydrogel in the absence or presence of polyP and then incubated for 5 days (Fig. 5II). In the absence of polyP within the hydrogel the surfaces of the cells are smooth and do not show any undulations in the morphology of their cells surfaces (Fig. 5IIA–C). In contrast, addition of polyP, as “Ca–polyP-NP”, induces a bulging of the cell surfaces (Fig. 5IID) and after inclusion of DEX and ascorbic acid, as “Ca–polyP/D-NP@polyP/AA-Coa”, the formation of crystallites was initiated (Fig. 5IIE), which progress also in size (Fig. 5IIF).

**Figure 6:**
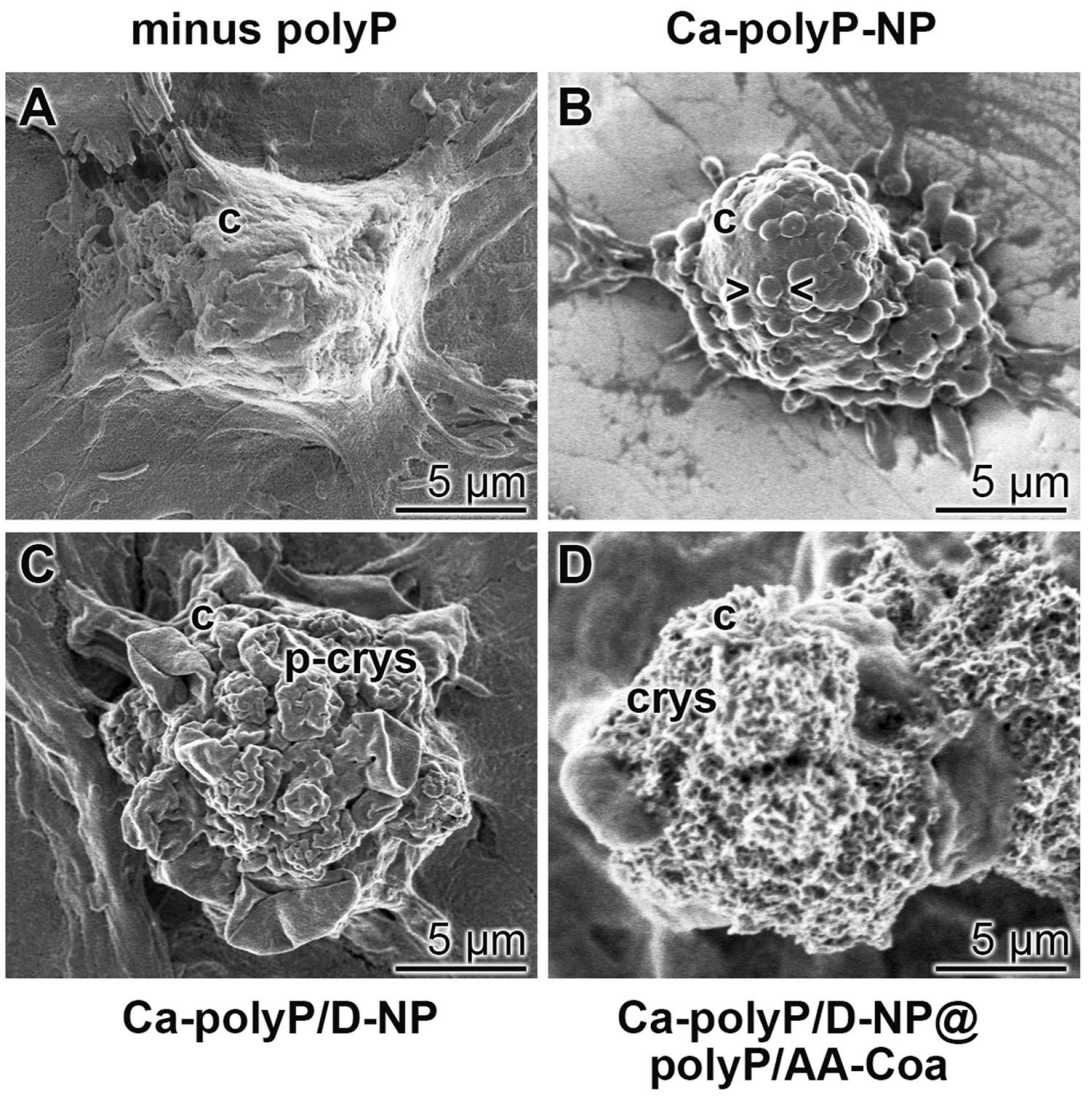
Change of the morphology of the SaOS-2 cell surface in dependence on the incubation condition within the alginate hydrogel after an incubation for 5 days. Representative images are shown; SEM. (**A**) In the absence of polyP in the gel the cell surface is smooth. This morphology alters after exposure of the cells to the gel containing polyP. (**B**) In gel containing “Ca–polyP-NP” the cell surface undulates >  < ; (**C**) after exposure to “Ca–polyP/D-NP” the cells start to form pre-crystallites (p-crys) and (**D**) after incubation with “Ca–polyP/D-NP@polyP/AA-Coa” the surface is studded with crystallites (crys).

A closer inspection of the cell surfaces was performed by high-resolution SEM (Fig. 6). The cells were grown for 5 days in the polyP-free hydrogel (Fig. 6A). Those cells show a smooth surface and no crystallite deposits. In contrast, the cells which are present in the hydrogel enriched with “Ca–polyP-NP” show distinct undulated cell surfaces (Fig. 6B), most likely structures caused by an accumulation of sub- membranous secretory vesicles, filled with pro-collagen^42^. Enrichment of the particles with DEX, as in “Ca–polyP/D-NP”, induces a progression of the pre-crystallite formation (Fig. 6C), while a final implementation also with ascorbic acid, as “Ca–polyP/D-NP@polyP/AA-Coa”, results in the formation of well-developed crystallites which are very numerous (Fig. 6D).

## Discussion

Polyphosphate (polyP) is a physiological inorganic polymer, synthesized within the cells in close association with the mitochondria and the adjacent acidocalcisomes [reviewed in: ref.^13^]. Its pivotal role in human metabolism becomes overt by the findings that in any repair process in the body, especially during reconstitution of osteomuscular defects or during wound healing, blood platelets accumulate at the sites of the defect and release polyP in the form of nanoparticles^43^. Those particles of polyP with divalent metal ions (Ca^2+^ or Mg^2+^) are formed intracellularly by the mitochondrial/acidocalcisomal complex and are secreted into the extracellular space by the platelets in the form of insoluble spherical nanoparticles. The biomimetic fabrication of those polyP nanoparticles has been achieved by using an over-stoichiometric ratio of the divalent cation to the phosphate units of the polyP polymer^20^. These bioimitated particles require an alkaline milieu (pH ~ 10) during their preparation. Released into the cytoplasm with of a pH near 7 the particles remain stabile until they come into contact with peptides/proteins which reduce their zeta surface potential and turn them to a coacervate phase^16^. During coacervation a temporal inclusion of metabolites needed by the cell can take place, which are re-supplied again upon demand. In the present study this transformation of linear polyP via Ca–polyP nanoparticles, enriched with a bioactive compound, to a Ca–polyP coacervate, likewise doped with a second active metabolite, was taken as a template to fabricate a core–shell system suitable for drug delivery. In the present study two metabolites causing osteogenic differentiation, dexamethasone (DEX) and ascorbic acid^22^, were encapsulated in the core–shell particles. In a related study, bioimitated granular platelet-sized polyP nanoparticles have been fabricated and packed into stabilized liposomes. In these 150 nm large particles polyP retains its functional activity on blood clotting^44^.

**Figure 7:**
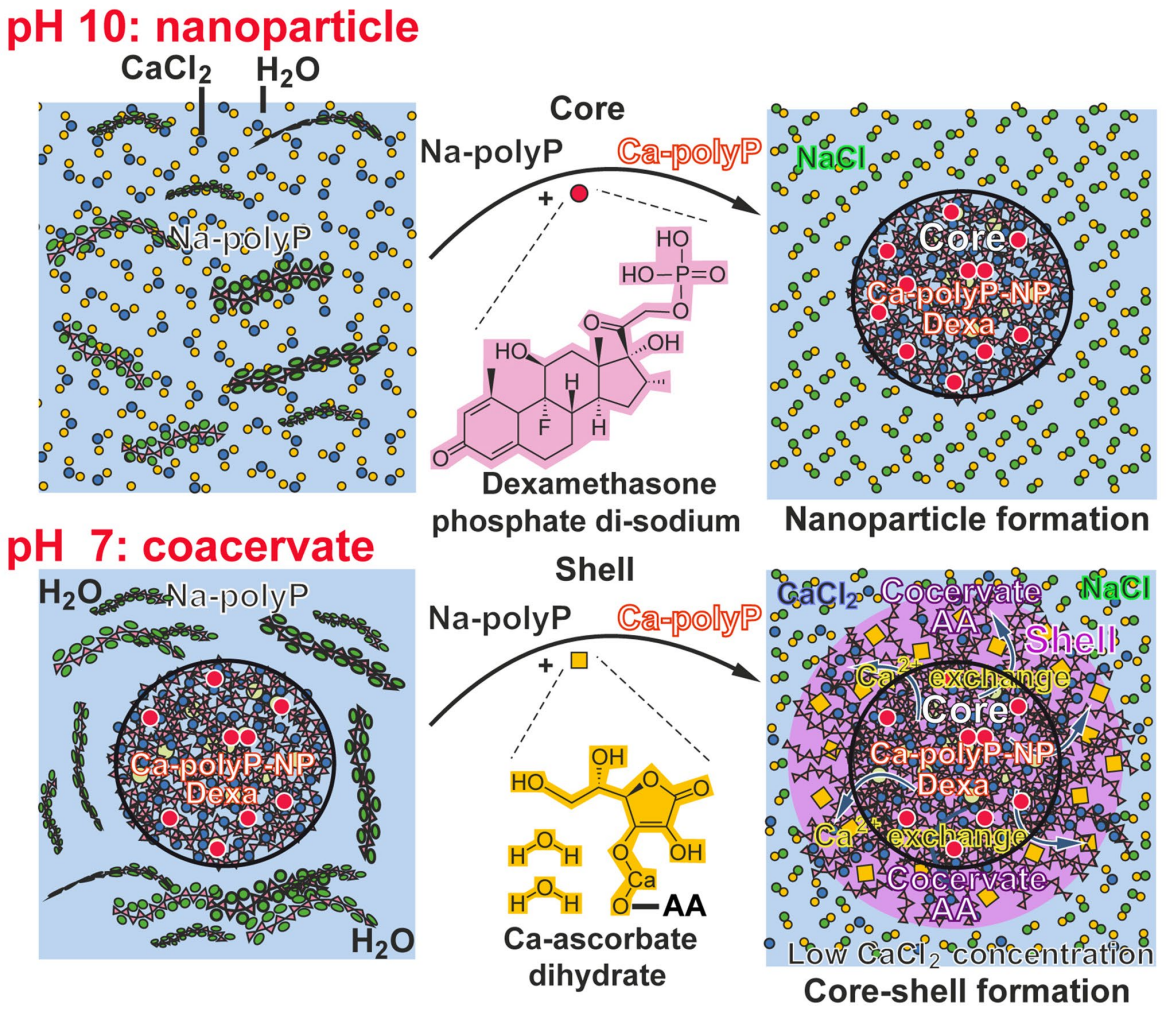
Formation of the polyP-based core–shell particles (scheme). Top: preparation of nanoparticles from Na–polyP and CaCl_2_ at pH 10. DEX, applied as DEX-P, was added to the reaction. Particle formation is based ionic interactions of the components. Bottom: Around the nanoparticles the coacervate formed by exposure of the nanoparticles to a Na–polyP solution was layered. The formation of the coacervate shell from the soluble Na–polyP was initiated by Ca^2+^ ions that are present in a stoichiometric surplus compared to the polyP phosphate units in the nanoparticle core. During the coacervation process at a pH of 7, these Ca^2+^ ions migrate from the core towards the Na–polyP rich environment. In addition, the Ca^2+^ ions that are linked to the calcium l-ascorbate dehydrate form ionic bridges to the polyanion.

It is straightforward to encapsulate bioactive compounds which are ionically charged into polyP nanoparticles. A scheme of the principle of the complete core–shell particle formation is given in Fig. 7. The limiting factor is that during fabrication of the particles the compound to be released must be stabile in the alkaline environment. In turn, the synthetic glucocorticoid DEX was supplied as dexamethasone 21-phosphate disodium (DEX-P) to the Na–polyP at pH 10. DEX remains stabile at this pH. To initiate the nanoparticle core formation, CaCl_2_ was added to an aqueous solution of soluble Na–polyP. The resulting Na–polyP nanoparticles, doped with DEX-P, “Ca–polyP/D-NP”, were washed and dried. The linkage between the components is based on an ionic bonding of the oppositely charged ions.

The determination of the distribution of the elements Ca and P in the nanoparticles revealed a stoichiometric surplus of Ca^2+^. Therefore, addition of CaCl_2_ to the system was not needed to allow the Ca–polyP coacervate phase to form. In this step the “Ca–polyP/D-NP” were suspended in an aqueous solution containing Na–polyP and calcium l-ascorbate dihydrate and incubated at a pH of 7. Ascorbic acid is stabile in this environment. During the following 30 min incubation period the nanoparticles became coated with a coacervate shell due to the migration of Ca^2+^ from the core into the shell region to form the Ca–polyP coacervate; “Ca–polyP/D-NP@polyP/AA-Coa”. The Ca^2+^ in the calcium l-ascorbate dihydrate chelates with the ascorbic acid and the polyP^45^.

The “Ca–polyP/D-NP@polyP/AA-Coa” particles are fairly stabile during a 12 h incubation period in saline, while in the presence of serum they transform rapidly into the functionally active coacervate. This result already proves their biological activity. This finding is also reflected in the release kinetics of DEX from the core–shell particles, which showed that less than 10% of DEX is released from the particles in saline during a 3 days treatment, while over 45% of the corticosteroid can be found in the surrounding serum enriched medium. This value is in the range also found for the release of DEX from poly(d,l-lactide-*co*-glycolide) particles^46^. In comparison, the ascorbic acid present in the shell of the core–shell particles is more readily liberated from the particles in the presence of serum, with a value of ~ 70% after 3 d, while less than 15% is released from the particles suspended in saline solution. This kinetics again meets published kinetics^47^ and reflects earlier findings showing that the coacervate phase of polyP is functionally more active, and in turn more dissolvable, compared to the nanoparticle form of polyP.

Cell biological studies revealed that the core–shell particles affect the cellular metabolic activity at concentrations higher than 100 µg mL^−1^. It remains to be studied if polyP, if encapsulated into particles causes apoptosis as described for chain-like polyP at high concentrations of 3 mM^48^. The core–shell particles fabricated here had been embedded into an alginate-based bio-ink in order to determine if SaOS-2 cells, exposed to those particles retain their mineralization activity also in this environment. As widely recognized these cells require for in vitro mineralization the components dexamethasone, ascorbic acid and phosphate^22^. As reported earlier^12^ polyP can substitute for β-glycerophosphate, since this polymer undergoes enzymatic hydrolysis to orthophosphate, which is needed as a phosphate source for mineralization and at the same time for the release of the metabolic energy required for mineral formation. In turn, SaOS-2 cells were incubated in an alginate hydrogel for 5 days in assays either without polyP or in the presence of “Ca–polyP-NP”, “Ca–polyP/D-NP”, or “Ca–polyP/D-NP@polyP/AA-Coa”. After staining with Alizarin Red the hydrogel was only slightly stained for mineralization in the assays with polyP or polyP and DEX, while an intensive staining was measured in the series with ascorbic acid. This result already shows that those particles that contain both DEX, ascorbic acid and polyP provide the optimal conditions for mineralization of the cells in vitro. A direct visualization of the minerals on the surface of the SaOS-2 cells was achieved by SEM imaging. The cells growing on the hydrogel showed extensive mineralization in vitro, only when all three components (DEX, ascorbic acid and polyP) were included into the growth system. Likewise, only if all three osteogenic components were present, an increased, in this case an even more pronounced, mineral formation was found if the cells had been embedded into the alginate-based hydrogel.

The core–shell particles developed here combine several advantageous properties that make them a promising tool not only for storage and delivery of drugs useful for bone regeneration and repair, but also in many other medical applications.

A striking feature of these particles is the fact that they respond both to the surrounding pH and peptides/proteins present in the environment that convert them into a functionally active coacervate state. Furthermore, based on previous results it can be expected that their integrity, if present in the coacervate state, can be influenced by the activity of polyP-metabolizing enzymes, with the ALP as the most relevant, both in tissue fluids and on the cell surface^13^.

There are many disorders which are associated with changes in pH, protein content/composition, or ALP activity over the course of the disease. For example, wound healing is associated with time-dependent changes in one or more of these parameters. The ALP activity has been shown to increase during wound healing, at the end of the inflammatory phase and with the progress of the granulation phase^49^. It has been shown that polyP nano/microparticles accelerate the wound healing process in both normal and diabetic mice^49^. Even ocular surface disorders and aging can be associated with characteristic changes in pH and ALP activity^50^, which might be relevant in view of the beneficial effects on cell viability/growth and migration of human corneal epithelial cells found for polyP and nano/microparticles formed by the polymer. The differential effects of the pH in the tissue environment on the osteoblast and osteoclast function have already been mentioned in the “Introduction”.

Another advantageous property of the core–shell particles is that, as shown here, they can be loaded with two (or presumably with even more) different drugs, in the inner Ca–polyP-NP core and the outer Ca–polyP-based shell, that will be released with different time kinetics due to the different responsiveness of the polyP in the Ca–polyP core and the surrounding shell to pH, protein and ALP activity. It should be noted that Ca–polyP nanoparticles are extremely stable and only slightly degradable by ALP. The polyP chains are only hydrolyzed at a significant extent by the enzyme after the particles have been converted into the coacervate phase^13^.

A limitation in the applicability of the method is the fact that immobilization of the drug requires the drug be either a (poly)cationic molecule that can bind directly to the polyP via ionic interaction, or a (poly)anion that can be immobilized via Ca^2+^ bridges. An example for the latter case is the bisphosphonates, a group of pyrophosphate-like molecules which are widely used in the treatment of bone tumors and osteoporosis^51^. Previously we have shown that the bisphosphonate zoledronic acid can be immobilized on Ca–polyP nanoparticles^12^. The particles showed both the morphogenetic effect of polyP and the cytostatic activity of zoledronic acid.

It should also be noted that the calcium ions used to prepare the Ca–polyP nanoparticles/coacervate are replaceable by other divalent cations such as Mg^2+^, Sr^2+^ or even trivalent (e.g., Gd^3+^)^13^ cations. As shown earlier, the exchange of the metal ion can lead to different properties of the polyP particles. It has been found that that Mg-polyP particles preferentially show regenerative activity on cartilage^12^, while Sr-polyP stimulates bone mineralization^14^. The novel principle described here (nanoparticle-coacervate conversion), which is the basis for the production of the Ca–polyP core–shell particles, should also be applicable for other divalent/trivalent cations. It is therefore expected that the developed technology will prove to be a versatile method for various applications in medical therapy.

## Conclusions

This study shows that the core–shell nanoparticles/coacervate method is a powerful system for the fabrication of drug-delivering core–shell particles that might have a great potential for bio-applications. Since both the synthesis of the nanoparticle core and the formation of the coacervate shell involves interactions between oppositely charged (poly)electrolytes in aqueous solution, a straightforward system for drug microencapsulation can be easily implemented if the components have ionic groups or coordinative potentials. The study presented here also contributes to the further development of colloidal engineering of nanoparticles for reversible and predictable systems for medical applications. Here the use of the core–shell particles formed from nanoparticles and coacervate for bone reconstitution and repair is documented. Because the coacervation process is controlled by Coulomb attractive forces and the entropy-driven release of counterions and is modulated by the main driving forces pH and ionic strength, which cause a change in the polyelectrolyte complexation, an individual adaptation of the hybrid core–shell particles to the existing tissue environment is possible. This accomplishment will deserve further patient-oriented elaboration. In addition, the strategy presented should also enable the fabrication of supramolecular assemblies by a templated, enzymatically directed organization of biological and technical materials, with the possibility of a spatio-temporal control. The construction of new synthetic super-protein assemblies with sophisticated functions from initially separated protein building blocks should be possible.
